# A rare case of retropharyngeal liposarcoma: a rare location of a rare diagnosis

**DOI:** 10.1093/jscr/rjad106

**Published:** 2023-03-07

**Authors:** Wajiha Arshad, Shahzaib Maqbool, Javeria A Kiany, Ali Raza, Umer Farooq, Qasim Ali, Ka Y Lee

**Affiliations:** Department of Surgical Unit II, Holy Family Hospital, Rawalpindi, Pakistan; Department of Surgical Unit II, Holy Family Hospital, Rawalpindi, Pakistan; Department of Surgical Unit II, Holy Family Hospital, Rawalpindi, Pakistan; Department of Surgical Unit II, Holy Family Hospital, Rawalpindi, Pakistan; Department of Surgical Unit II, Holy Family Hospital, Rawalpindi, Pakistan; Department of Surgical Unit II, Holy Family Hospital, Rawalpindi, Pakistan; Department of Health Sciences, Mid Sweden University, Ostersund 83125, Sweden

**Keywords:** Liposarcoma, retropharyngeal space, retropharyngeal liposarcoma

## Abstract

We report a case of retropharyngeal liposarcoma in a 53-year-old female, who had complaints of neck swelling accompanied with dysphagia, orthopnea and dysphonia. Clinical examination revealed huge multinodular swelling in front of neck with bilateral extension, more prominent on left side and moving with deglutition. The diagnosis of retropharyngeal liposarcoma was established following CT scan, MRI and incisional biopsy. Surgical excision of mass along with near total thyroidectomy was performed. Postoperative hospital stay was uneventful. She remained well in follow-up period of 1 year as well. In conclusion, retropharyngeal liposarcoma is a rare tumor. A review of the literature explores the reasons behind the late presentation as well as the difficulties in diagnosis and treatment of this rare tumor.

## INTRODUCTION

Liposarcoma is a common type of soft tissue sarcoma that accounts for about 20% of all adult sarcomas. The common place for liposarcoma to develop is in deep soft tissues of lower limbs and retroperitoneal parts, accounting for 24 and 45% of limb sarcomas and retroperitoneal soft tissue sarcomas, respectively [1, 2]. Atypical lipomatous tumors are locally aggressive mesenchymal lipogenic tumors that arise infrequently in head and neck region. The word atypical lipoma was introduced to depicts the benign course of well-differentiated liposarcomas [3]. Very few cases of liposarcomas of retropharyngeal space are reported in the literature. Herein, we reported a case of well-differentiated/atypical lipomatous tumor of retropharyngeal space in a 53-years old female with no known comorbidities.

## CASE REPORT

A 53-year-old female with no comorbids presented to outdoor surgical department of Holy Family Hospital, Rawalpindi, Pakistan with complaints of swelling in front of neck for past 1.5 years that was enlarging progressively, along with dysphagia for last 3 months with more predilection for solid, orthopnea for 2 months and dysphonia for 1 month. There was no history of any swelling in this or any other location in the past. There was no history of smoking or any exposure to radiation.

**Figure 1:**
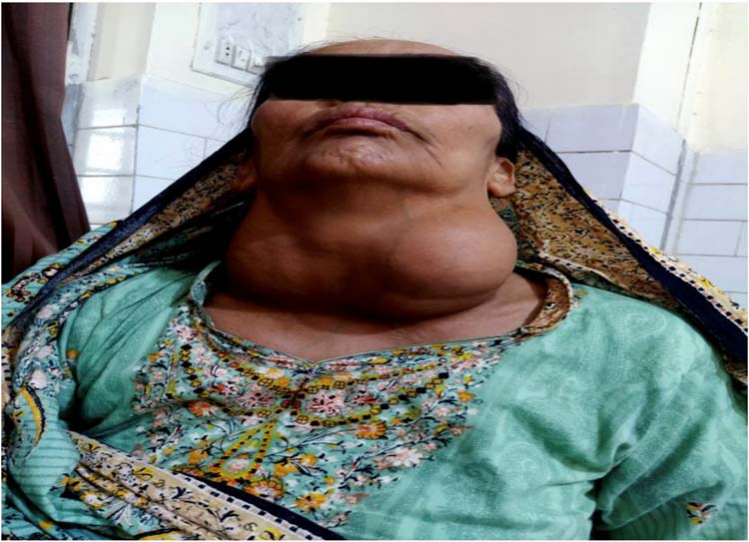
Preoperative picture showing the gross presentation of neck mass occupying bilateral cervical area with more bulk in left cervical region.

Clinical examination revealed stridor along with huge multinodular swelling ~15 × 10 cm in front of neck with bilateral extension, more prominent on left side as shown in Fig. 1. It moves with deglutition and overlying skin revealed prominent veins. Trachea was not palpable, and no cervical lymphadenopathy was appreciated. Indirect laryngoscopy showed bilateral mobile vocal cords with no visible growth.

**Figure 2:**
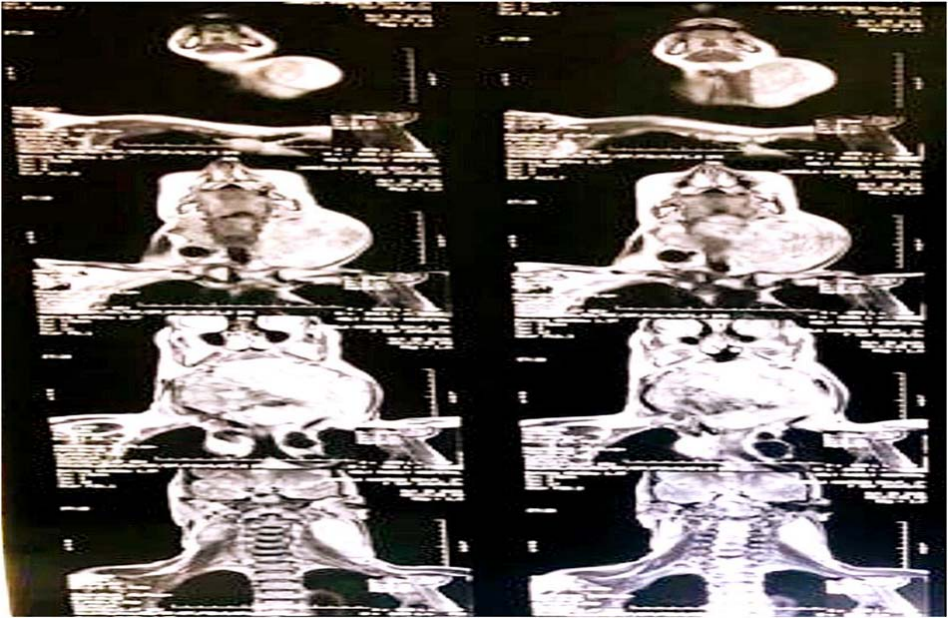
CT scan demonstration a well-defined mass in retropharyngeal space measuring 9 × 15 × 11 cm in various dimensions and chunk of calcific foci occupying most of the left retropharyngeal space.

**Figure 3:**
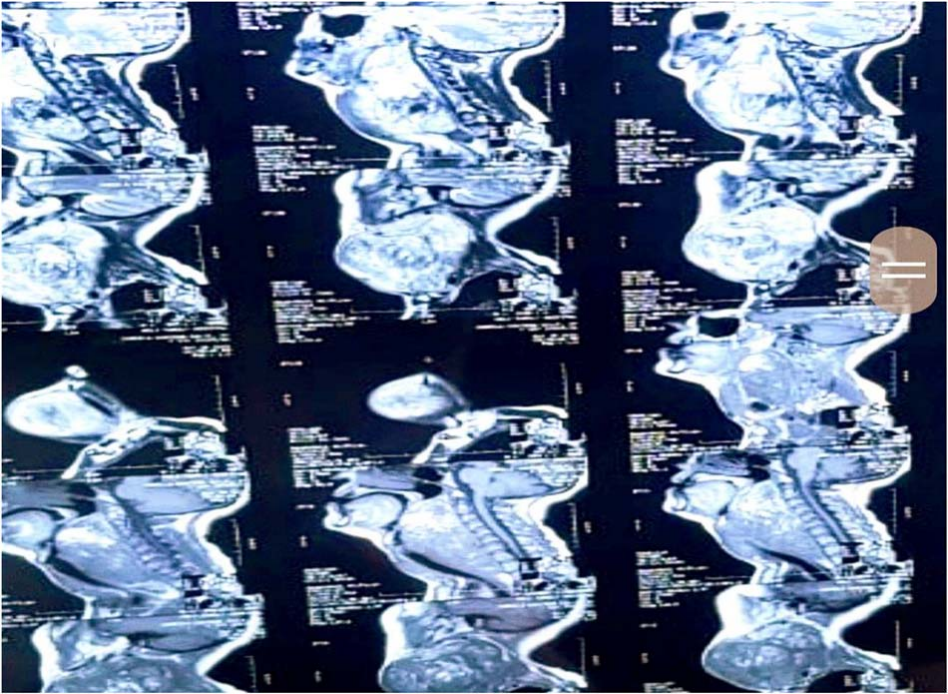
MRI scan demonstration of mass location and extension depicted at various levels of scan measuring 10 × 13.5 × 12.5 cm in size and appearing heterogeneously isointense on T1W1 scan and heterogeneously hyperintense on T1WI and STIR sequence.

Upon radiological investigations (X-ray, ultrasound, CT scan and MRI), a provisional diagnosis of retropharyngeal differentiated liposarcoma was made. On USG, the thyroid enlargement along with solid mass measuring 37 × 47 mm of size in cervical region was observed. The thyroid enlargement was attributed as multinodular goiter. On CT scan, a mass measuring 9 × 15 × 11 cm with bulging into left cervical region with extension into carotid space causing stretching of carotid vessels was observed. On CT scan analysis, a single enlarged lymph node in Para tracheal region was also observed. The CT scan findings are given in Fig. 2. MRI analysis was also showing a left retropharyngeal mass with calcification measuring about 10 × 13.5 × 12.5 cm in different dimensions pushing the pharynx, larynx and trachea anteriorly and a provisional diagnosis of dedifferentiated liposarcoma was made. MRI imaging of the patient is given in Fig. 3.

**Figure 4:**
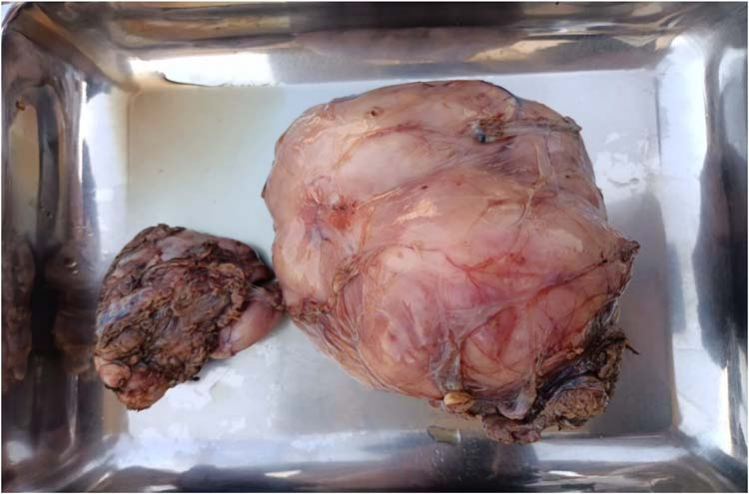
Excised neck mass along with thyroid gland. The larger mass is representative of Liposarcoma and smaller one is thyroid gland.

FNAC was attempted twice but it reveals hemorrhage only and was inconclusive. Incisional biopsy was done, which revealed atypical spindle cell lipomatous tumor. After complete workup, she underwent excision of mass along with near total thyroidectomy and tracheostomy and excised mass was sent for histopathology, which confirmed it to be atypical lipomatous tumor (well-differentiated liposarcoma). Postoperative period was uneventful, and patient remained stable during ward stay and was later discharged with follow-up. She was followed up to 1 year, no recurrence seen. The excised mass along with respect thyroid gland is given in Fig. 4.

## DISCUSSION

Liposarcoma is among the most common types of soft tissue sarcoma in adults, and usually occurs in the lower extremities, retroperitoneum, perinephric space, mesentery or shoulders. The incidence of liposarcoma in the head and neck region, at ~1.8–6.2%, is extremely low [4]. Their mortality ranges from 28 to 50%, and they grow quicker and develop metastases, mostly to the lungs [5].

The most common histological type is the well-differentiated one (this type includes the adipocytic, sclerosing and inflammatory subtype), accounting for 40–50% of all types, whereas the most infrequent type is pleomorphic liposarcoma [6]. According to a study published by Karamitsou *et al*. [7], ~10% of well-differentiated liposarcomas have the potential to transform into dedifferentiated liposarcomas, which is linked to a more aggressive clinical behavior. Dedifferentiated liposarcomas usually present as undifferentiated pleomorphic or spindle cell sarcomas, and they can develop either on their own or in association with an existing well-differentiated liposarcoma [7]. FISH analysis for MDM2 (and/or CDK4) amplification is a helpful diagnostic tool for identifying dedifferentiated liposarcomas. Surgical removal is the primary treatment for localized dedifferentiated liposarcomas, although in some rare cases, radiation therapy or systemic treatments may be combined with surgery [8]. Although WDLS has a low metastatic potential and a good prognosis, incomplete resection may result in local recurrence, which can lead to dedifferentiation and a less favorable outcome. The 30 pathologically confirmed cases of liposarcoma of the head and neck region identified between 1945 and 2005 represented ~2% of all sarcomas of the head and neck region seen during that period. Of 30 liposarcomas in our series, seven (23%) were well differentiated, and one case was reported with location of primary liposarcoma as retroesophageal/retropharyngeal [9, 10].

In most cases, the liposarcoma occurred in patients > 60 years of age. Apart from their genetic predisposition, well-known risk factors for soft-tissue sarcomas are iatrogenic factors such as a chronic lymphedema and previous radiation; however, liposarcomas seem to be only rarely associated with these [5]. Although rare, there have been reports of these neoplasms arising from preexisting lipomas [11]. It is important to note that liposarcomas do not develop from lipomas, which are completely benign [12].

Patients with liposarcoma are usually asymptomatic, however; they can produce pressure symptoms, such as dysphagia, dyspnea and dysphonia. The retropharyngeal space lies between the buccopharyngeal fascia and the prevertebral fascia and extends from the skull base to the mediastinum [9]. Disease processes in the retropharyngeal space are relatively uncommon; however, they assume greater significance because of the proximity of the retropharyngeal space to the airway and because of the inability to examine this space clinically [13, 14].

The diagnosis of a liposarcoma is typically delayed secondary to its indolent, asymptomatic course. Imaging modalities aid the clinician in diagnosing liposarcomas, and the most utilized are the MRI and CT scans [11]. The amount of radiologically identifiable fat at MR is variable, but a well-differentiated liposarcoma will generally demonstrate at least 75% of adipose tissue. CT and MR also tend to reflect the degree of tumor differentiation, the more differentiated the tumor, the more the image appearance will approach that of adipose tissue [15]. The gold standard in diagnosing a liposarcoma remains the biopsy. This can be done through fine needle aspiration and incisional or excisional biopsy [11].

Although surgery with negative margins is very well documented as the treatment of choice for all histopathological subtypes of head and neck liposarcomas, routine regional lymph node dissection is not generally recommended, as node metastases of head and neck liposarcomas are quite rare [11]. Postoperative radiotherapy is advocated by most authors, especially in patients with large tumors, high-grade lesions, local extension and incomplete resection-positive margins. Postoperative adjuvant radiotherapy appears to decrease the rate of local recurrence (from 60 to 40%) but it does not seem to improve the overall survival rate or the rate of metastasis [11]. A well-differentiated liposarcoma has been reported to have a 50% recurrence rate with no risk of distant metastasis and an excellent 5-year survival rate (75–100%) [12]. Whereas the 5-year survival rate for all liposarcomas of the head and neck has been reported at 45–65% [16].

## CONFLICT OF INTEREST STATEMENT

None declared.

## FUNDING

None.

## DATA AVAILABILITY

Data supporting the conclusions are included in the article.

## References

[1] Gamboa AC, Gronchi A, Cardona K. Soft-tissue sarcoma in adults: an update on the current state of histiotype-specific management in an era of personalized medicine. CA Cancer J Clin 2020;70:200–29. 32275330 10.3322/caac.21605

[2] Yang L, Chen S, Luo P, Yan W, Wang C. Liposarcoma: advances in cellular and molecular genetics alterations and corresponding clinical treatment. J Cancer 2020;11:100. 31892977 10.7150/jca.36380 PMC6930414

[3] Narayana ML, Mohiyuddin SA, Viswambharan V, Gaur U, Swethadri GK. A rare case of atypical lipoma in retropharyngeal space and review of literature. Int J Otorhinolaryngol Head Neck Surgery 2019;5:1708.

[4] Yang T, Xiao L, Ren H. A well-differentiated liposarcoma of the prevertebral space: a case report. Transl Cancer Res 2021;10:3600. 35116663 10.21037/tcr-21-143 PMC8797681

[5] Guarda V, Pickhard A, Boxberg M, Specht K, Buchberger AM. Liposarcoma of the thyroid: a case report with a review of the literature. Eur Thyroid J 2018;7:102–8. 29594062 10.1159/000486333 PMC5869545

[6] Papacharalampous GX, Kikidis D, Vasileiou A, Bousiotou A, Chrysovergis A. Liposarcoma of the nasopharynx: diagnosis and management of a rare diagnostic entity. Case Rep Med 2012;2012:314697. 10.1155/2012/314697 PMC3318223 22489241

[7] Karamitsou P, Skliris JP, Karamitsou A, Forozidou E, Poutoglidis A. A case of dedifferentiated laryngeal Liposarcoma with metachronous transformation into a neoplasm with myxofibrosarcomatous elements. Cureus 2022;14:e30901. 10.7759/cureus.30901 PMC9709908 36465752

[8] Nishio J, Nakayama S, Nabeshima K, Yamamoto T. Biology and management of dedifferentiated liposarcoma: state of the art and perspectives. J Clin Med 2021;10:3230. 34362013 10.3390/jcm10153230 PMC8348700

[9] Ikeda M, Fujii S, Morishita Y, Hayashi R. Value of intraoperative pathological diagnosis in decision-making regarding resection of well-differentiated retropharyngeal liposarcoma: a case report. Int J Surg Case Rep 2021;88:106466. 34653896 10.1016/j.ijscr.2021.106466 PMC8521110

[10] Davis EC, Ballo MT, Luna MA, Patel SR, Roberts DB, Nong X, et al. Liposarcoma of the head and neck: the University of Texas MD Anderson Cancer Center experience. Head Neck 2009;31:28–36. 18767171 10.1002/hed.20923

[11] Gleinser DM, Font JP, Clement CG, Mohammed BS, Underbrink MP. Primary myxoid liposarcoma of the supraglottic larynx. Rare Tumors 2010;2:112–4. 10.4081/rt.2010.e41 PMC2994524 21139957

[12] Zafar R, Wheeler Y. Liposarcoma. 2022. https://pubmed.ncbi.nlm.nih.gov/30855853/ (3 November 2022, date last accessed). 30855853

[13] Shi H, Wang S, Wang P, Yu Q. Primary retropharyngeal synovial sarcoma. Am J Neuroradiol 2009;30:811–2. 18854438 10.3174/ajnr.A1333 PMC7051765

[14] He JG, Jiang H, Yang BB, Lin PF. Liposarcoma of the retropharyngeal space with rapidly worsening dyspnea: a case report and review of the literature. Oncol Lett 2013;5:1939–42. 23833671 10.3892/ol.2013.1310 PMC3700895

[15] Rubin DJ, Lee SL. Anterior neck lipoma mimicking a goiter. Healio 2009. https://www.healio.com/news/endocrinology/20120325/anterior-neck-lipoma-mimicking-a-goiter (3 November 2022, date last accessed).

[16] Gundelach R, Ullah R, Coman S, Campbell K. Liposarcoma of the retropharyngeal space. J Laryngol Otol 2005;119:651–4. 16102226 10.1258/0022215054516296

