# Correction to: A rare case of retropharyngeal liposarcoma: a rare location of a rare diagnosis

**DOI:** 10.1093/jscr/rjad313

**Published:** 2023-05-15

This is a correction to: Wajiha Arshad, Shahzaib Maqbool, Javeria A Kiany, Ali Raza, Umer Farooq, Qasim Ali, Ka Y Lee, A rare case of retropharyngeal liposarcoma: a rare location of a rare diagnosis, *Journal of Surgical Case Reports*, Volume 2023, Issue 3, March 2023, rjad106, https://doi.org/10.1093/jscr/rjad106

In the originally published version of this manuscript, author Ka Y Lee's affiliation, Department of Health Sciences, Mid Sweden University, Ostersund 83125, Sweden, was inadvertently omitted.

This error has been corrected.

